# Integrated Serum and Urinary Metabolomics Reveal Distinct Signatures of Kidney Graft Function—A Single-Center Cohort Study

**DOI:** 10.3390/biomedicines14081875

**Published:** 2026-08-21

**Authors:** Cezar Valeriu Băluță, Lucian Siriteanu, Ionuț Nistor, Luminița Voroneanu, Simona Mihaela Hogas, Andreea Covic, Călin Namolovan, Raluca Erika Irimie Băluță, Radu Gavril, Calin Deleanu, Alina Nicolescu, Mehmet Kanbay, Adrian Covic

**Affiliations:** 1 Grigore T. Popa University of Medicine and Pharmacy, 700115 Iași, Romania; 2 “Costin D. Nenitescu” Institute of Organic and Supramolecular Chemistry, 060023 Bucharest, Romania; 3 “Petru Poni” Institute of Macromolecular Chemistry, 700487 Iași, Romania; 4 Department of Medicine, School of Medicine, Koç University, Istanbul 34450, Türkiye

**Keywords:** graft function, kidney transplantation, metabolomics, NMR spectroscopy, proteinuria

## Abstract

**Background/Objectives**: Kidney transplantation remains the gold standard treatment of end-stage kidney disease; however, its recipients exhibit mixed clinical trajectories, with variable renal function and proteinuria evolution over time. Currently used methods to screen these changes are associated with possible adverse effects and limitations. **Methods**: In this single-center cohort study, 174 adult kidney transplant recipients were enrolled between January 2024 and January 2025 and followed for 12 months. Patients underwent serum and urinary metabolomic profiling using nuclear magnetic resonance (NMR) spectroscopy. Furthermore, clinical and biological data were collected, allowing for assessment of changes in renal function and proteinuria, with patient stratification based on outcome trends. **Results**: Serum metabolomic profiles were linked to renal outcomes. For ΔeGFR, valine differed between patients with positive and negative trajectories, while GlycA, GlycB and Glyc/SPC were associated with ΔeGFR in adjusted models. For Δproteinuria, GlycA, VLDL particles and triglycerides showed correlations, while glucose differed between patients with improving and worsening proteinuria. In adjusted models, isoleucine, alanine, GlycA and Glyc/SPC were associated with Δproteinuria. Urinary metabolomic profiles showed fewer associations. Dimethylamine and urinary creatinine were associated with ΔeGFR in adjusted models, while acetic acid differed between proteinuria groups. No urinary metabolite was associated with Δproteinuria after adjustment. **Conclusions**: Overall, metabolomic changes were connected with renal outcomes in kidney transplant patients, with distinct but partially overlapping patterns for ΔGFR and Δproteinuria. Serum findings were more consistent, while urinary associations were more limited. Further longitudinal and externally validated studies are required to confirm these observations and explore the potential of this metabolomic approach in the search for novel biomarkers.

## 1. Introduction

Chronic kidney disease represents one of the leading causes of death worldwide, ranking ninth in the top 10 causes of death, and registering an increase of over 90% between 2000 and 2021 [1]. Once patients reach end-stage, kidney replacement therapy is required. Among different available options, kidney transplantation is the most efficient and desired [2].

Conventional allograft evaluation is performed by routine creatinine and proteinuria measurement, but these biomarkers are limited and imperfect, usually signaling graft damage with a degree of latency [3]. The current gold standard, graft biopsy, is linked with possible serious adverse effects [4]. Therefore, there is a need for new, more reliable biomarkers which can detect early graft injury. Metabolomics, the science that studies small molecules in different biological samples, may be used in the search for said new biomarkers [5]. Metabolomic profiling can be performed on serum and urine samples and may offer additional information without the need for more invasive procedures such as graft biopsy. If specific metabolic patterns associated with worse renal outcomes are identified and validated, metabolomics could help identify patients at higher risk who may benefit from closer follow-up. Serum and urine may also provide complementary information on systemic and renal metabolic changes.

In this study we performed serum and urine metabolomic analysis along with routine ambulatory evaluation (which is performed for all our kidney transplant patients) at baseline. We were thus able to generate an initial metabolomic profile that was associated with graft function. We prospectively followed this cohort for 12 months, after which a new regular ambulatory biological assessment was performed. This design provided us with an initial metabolomic pattern, allowing us to explore how this early profile could be linked to future kidney graft function.

The aim of this study was to identify baseline serum and urinary metabolomic signatures associated with changes in graft function and proteinuria over a 12-month follow-up period. We started from the presumption that baseline serum and urinary metabolomic profiles would be associated with 12-month changes in graft function and proteinuria.

## 2. Materials and Methods

The study was conducted in accordance with the ethical principles of the Declaration of Helsinki. Ethical approval for patient enrolment and study procedures was obtained from the Ethics Committee of the “Dr. C. I. Parhon” Clinical Hospital (approval no. 1799, February 2024). The study was subsequently reviewed and approved by the Ethics Committee of the “Grigore T. Popa” University of Medicine and Pharmacy, Iași (approval no. 725, 2 March 2026). We conducted a single-center observational study of adult kidney transplant recipients. A total of 174 patients were included in the study between January 2024 and January 2025. No formal a priori sample size calculation was performed; as this was an exploratory study, all eligible patients who consented to participate were enrolled in the specified period. Study participants were recruited from the Transplant Center of “Dr. C.I. Parhon Hospital”, Iași, Romania. All participants were followed for 12 months after enrolment, with outcome assessment performed at the end of the follow-up period. For the last enrolled patient, follow-up ended in January 2026. We enrolled eligible kidney transplant patients attending routine ambulatory check-ups; follow-up consisted of scheduled 12-month ambulatory reassessment. Inclusion criteria were kidney transplant recipients with a minimum of 6 months after transplantation. Exclusion criteria were multiple organ transplantation, antibiotic use in the past 3 months, inflammatory bowel disease, inability to provide signed consent, advanced neuropsychiatric disease, and patient refusal. All patients provided written informed consent. Because serum and urine metabolomic datasets were not available for all patients, and some regression models required complete-case observations, the effective sample size varied across analyses. Serum analyses were performed in patients with available serum data, whereas urinary analyses were restricted to those with available urinary metabolomic data. The number of patients included in each analysis is indicated where relevant.

Primary outcomes were 12-month ΔGFR (defined as 12 month eGFR minus baseline eGFR) and Δproteinuria (defined as the baseline value minus 12-month proteinuria). Relevant data (demographic, transplant-related, medical history, comorbidities) were obtained from medical records and routine ambulatory assessments.

Immunosuppressive treatment followed the local transplant protocol. Basiliximab was used for induction in patients with low immunological risk, while rabbit anti-T-lymphocyte globulin was used in selected patients with higher immunological risk. Maintenance treatment generally included tacrolimus or cyclosporine, mycophenolate and corticosteroids. Tacrolimus was the preferred calcineurin inhibitor, with trough levels adjusted according to time from transplantation and individual risk. Calcineurin inhibitor exposure and corticosteroid doses were reduced over time when clinically appropriate. Sirolimus-based regimens were used only in selected patients.

### 2.1. Collection of Blood and Urine Samples

Blood samples were collected in the morning, after a 10 h fasting period, by trained personnel according to standardized procedures, from a peripheral vein into LPS-free tubes, thereby minimizing potential risk of measurement bias. Immediate centrifugation at 4 °C (30 min at 4000 rpm) was performed to separate plasma and serum components, followed by serum storage in 2 mL cryotubes at −40 °C until analysis. Urine was collected in specific specimen containers, from the first morning urine after strict local hygiene, and was stored in 2 mL cryotubes at minus 40 °C until analysis. Biological parameters were measured in the hospital laboratory using standard methods; eGFR was calculated using the 2021 CKD-EPI creatinine equation [6]. The same procedures were applied to all participants.

### 2.2. Metabolomic Determination

Using nuclear magnetic resonance (NMR), we performed metabolomic profiling and quantification. In serum, 21 metabolites and 118 lipoproteins were quantified, including triglycerides, cholesterol, apolipoproteins A1, A2 and B100, major HDL, IDL, LDL and VLDL fractions, glycoproteins (Glyc, GlycA and GlycB), and the supramolecular phospholipid signal. A full description of the lipoprotein parameters, including the subfraction densities, has been previously reported [7].

Serum samples were thawed at room temperature and mixed in a 1:1 ratio with a phosphate buffer solution before acquisition at 600 MHz on a Bruker Avance Neo spectrometer following standard operating procedures. A targeted analysis of urinary creatinine and 12 urinary metabolites (dimethylamine, alanine, glycine, proline betaine, valine, hippuric acid, citric acid, formic acid, succinic acid, acetoacetic acid, acetone, and D-glucose) was also performed using NMR and quantification of urinary metabolites was expressed relative to urinary creatinine. Before use, the NMR tubes were checked as previously described [8].

Continuous variables were tested for normality and analyzed according to their distribution. Baseline and 12-month clinical and laboratory values were compared using paired data. A paired t test was used for normally distributed differences and the Wilcoxon signed-rank test for non-normal differences. For each parameter, only patients with data available at both time points were included. ΔeGFR was calculated as eGFR at 12 months minus baseline eGFR, while Δproteinuria was calculated as baseline proteinuria minus proteinuria at 12 months. Positive values therefore indicated improvement for both outcomes. Spearman correlation was used to examine the relationship between baseline metabolite levels and changes in eGFR and proteinuria. Metabolite levels were also compared between patients with positive and negative ΔeGFR and between those with improving and worsening proteinuria using the Mann–Whitney U test. Patients with no change in the respective outcome were excluded from these comparisons.

Linear regression was used to further assess the association between baseline metabolites and ΔeGFR or Δproteinuria. Each metabolite was analyzed in a separate model adjusted for age, sex, baseline eGFR and transplant vintage. Transplant vintage was defined as the time from the most recent kidney transplantation to baseline sample collection. For ΔeGFR, an additional analysis was performed including calcineurin inhibitor class, tacrolimus or cyclosporine, in the model. Patients receiving neither drug were excluded from this analysis. Regression coefficients are reported as unstandardized β values with 95% confidence intervals. Analyses were performed using available complete cases, without imputation of missing clinical or covariate data. All tests were two-sided and *p* < 0.05 was considered nominally significant. No correction for multiple comparisons was applied because the analyses were exploratory. Statistical analyses were performed using Jamovi version 2.6 (https://jamovi.org).

Metabolomic data were processed in MetaboAnalyst using a standardized workflow. Missing values were replaced with one-fifth of the minimum positive value using limit-of-detection-based imputation. Low-variance features were filtered using an interquartile range-based method, removing 25% of variables. For multivariate analysis, data were additionally normalized by sum, log10-transformed to reduce skewness and stabilize variance, and autoscaled to ensure equal contribution of metabolites to downstream analyses.

After preprocessing, multivariate analyses were conducted, including principal component analysis (PCA), partial least squares discriminant analysis (PLS-DA), variable importance in projection (VIP) scores, and heatmap analysis. PLS-DA model performance was not externally validated and should therefore be interpreted cautiously due to the potential risk of overfitting (additional NMR sample preparations and further details are provided in the Supplementary Material). This study was reported in accordance with the Strengthening the Reporting of Observational Studies in Epidemiology (STROBE) guidelines for cohort studies.

## 3. Results

### 3.1. Baseline Characteristics

Demographic characteristics are presented in Table 1, while paired clinical and laboratory values at baseline and 12 months are shown in Table 2.

A total of 174 kidney transplant recipients were included, of whom 110 (63.2%) were male. Median age was 49 years (IQR 39–57). Overall, kidney function remained stable during follow-up, with no significant differences in serum creatinine, eGFR, urea or proteinuria between baseline and 12 months. Nominal differences were found for hemoglobin, white blood cell count, fasting glucose and uric acid, while the other laboratory parameters remained similar. The number of paired observations differed between parameters because of missing data.

### 3.2. Serum Metabolomics, ΔGFR and ΔProteinuria

Based on the change in eGFR at 12 months, 89 patients had a positive trajectory, 78 had a negative trajectory and 7 had no change. Proteinuria improved in 22 patients and worsened in 30, while 1 patient had unchanged values. Proteinuria was absent at both baseline and 12 months in 115 patients, and data were missing for 6 patients. Patients with unchanged values were excluded from the respective group comparisons. Serum and urinary metabolites were then analyzed in relation to both continuous changes and outcome trajectories. The main findings are summarized in Table 3 and described separately for serum and urine below.

### 3.3. Serum ΔGFR

Serum metabolites showed several associations with changes in eGFR at 12 months. Valine levels differed between patients with positive and negative ΔeGFR trajectories, with a Mann–Whitney *p*-value of 0.033. Borderline differences were also found for tyrosine and lactate, with *p*-values of 0.052 and 0.058. No significant Spearman correlations were observed. After adjustment for age, sex, baseline eGFR and transplant vintage, GlycA was associated with ΔeGFR with β 15.07, 95% CI 2.13 to 28.00 and *p* 0.023. Similar associations were found for GlycB with β 32.85, 95% CI 0.62 to 65.08 and *p* 0.046 and for Glyc/SPC with β 8.05, 95% CI 0.03 to 16.07 and *p* 0.049. The results remained similar after additional adjustment for calcineurin inhibitor class. Pyruvate, tyrosine, IDL particle number, lactate, acetic acid and creatine had VIP scores above 1.

### 3.4. Serum ΔProteinuria

Several serum metabolites were associated with changes in proteinuria. GlycA showed a positive correlation with Δproteinuria with Spearman ρ 0.319 and *p* 0.023. VLDL particle number and triglycerides showed negative correlations, with ρ −0.283 and *p* 0.044 for VLDL and ρ −0.277 and *p* 0.050 for triglycerides. Glucose levels also differed between patients with improving and worsening proteinuria, with a Mann–Whitney *p*-value of 0.038. In the adjusted regression analysis, isoleucine was negatively associated with Δproteinuria with β −18.28, 95% CI −32.39 to −4.17 and *p* 0.012. Alanine showed a similar negative association with β −3.77, 95% CI −7.25 to −0.28 and *p* 0.035. Positive associations were found for Glyc/SPC with β 1.74, 95% CI 0.23 to 3.25 and *p* 0.025 and for GlycA with β 2.95, 95% CI 0.25 to 5.64 and *p* 0.033. VIP scores above 1 were found for tyrosine, 3 hydroxybutyrate, triglycerides, VLDL particle number and succinic acid.

### 3.5. Urine ΔGFR

Urinary metabolite associations with changes in eGFR were more limited. Dimethylamine showed a weak positive correlation with ΔeGFR with Spearman ρ 0.156 and *p* 0.047. Citric acid showed a borderline difference between patients with positive and negative ΔeGFR trajectories, with a Mann–Whitney *p*-value of 0.080. In the adjusted regression analysis, dimethylamine was associated with ΔeGFR with β 23.05, 95% CI 8.06 to 38.03 and *p* 0.003. Urinary creatinine was also associated with ΔeGFR with β 0.61, 95% CI 0.15 to 1.07 and *p* 0.010. Citric acid remained borderline with *p* 0.066. The associations for dimethylamine and urinary creatinine remained similar after additional adjustment for calcineurin inhibitor class. Citric acid, formic acid and acetic acid had VIP scores above 1.

### 3.6. Urine ΔProteinuria

Only a few associations were found between urinary metabolites and changes in proteinuria. Acetic acid levels differed between patients with improving and worsening proteinuria, with a Mann–Whitney *p*-value of 0.031. No significant Spearman correlations were found. None of the urinary metabolites remained significantly associated with Δproteinuria in the adjusted regression analysis. Acetic acid, glycine and glucose had VIP scores above 1.

## 4. Discussion

We had hypothesized that baseline serum and urinary metabolomic profiles would be associated with 12-month changes in graft function and proteinuria. In this study, metabolomic changes linked to renal outcomes were multifactorial, with ΔGFR evolution being connected to inflammatory and energy-related pathways and proteinuria progression to lipid and amino acid metabolism.

In this study metabolomic changes that were linked to ΔGFR were subtle and involved multiple metabolites rather than a single one. Univariate analysis found few significant associations. Valine differed between patients with positive and negative ΔGFR trajectories, while tyrosine and lactate showed borderline differences. In multivariable regression, GlycA, GlycB and Glyc/SPC were associated with ΔGFR. A broader metabolomic pattern linked to ΔGFR is suggested by the fact that no single metabolite dominated the effect. PCA supports this, as it shows extensive overlap between groups, with a weak discrimination by PLS-DA. VIP analysis highlighted pyruvate, tyrosine, IDL particle number, lactate, acetic acid and creatine as the main contributors to ΔGFR change. These results imply that the renal function evolution is influenced by a complex systemic process that involves energy and inflammatory pathways.

Compared to the published literature, our findings show partial concordance, though differences in study design, endpoints, and methodology limit direct overlap to a degree. Both our data and previous studies show an implication of amino acid and organic acid metabolism in kidney dysfunction. Baranovićová et al. [9], in a 2022 study, identified that plasma levels of tyrosine fall and phenylalanine rises as kidney function worsens and at the same time acetate and citrate levels increase with lower GFR. Our study also identified tyrosine and acetic acid among the VIP contributors, but neither remained significant in the adjusted models. Lactate and pyruvate were also highlighted by VIP analysis while Baranovićová et al. found no such correlation. Bassi et al. [10] linked worsening graft function with increases in serum tryptophan, glutamine, and ADMA/SDMA and corresponding urine increases in histidine, dopamine, DOPA, carnosine, and ADMA/SDMA. We did not directly measure these metabolites, but our results underlined amino acid dysregulation.

Proteinuria evolution showed a similar but distinct metabolic profile. GlycA was positively correlated with Δproteinuria, while VLDL particle number and triglycerides showed negative correlations. Glucose also differed between patients with improving and worsening proteinuria. In multivariable regression, isoleucine and alanine were negatively associated with Δproteinuria, while GlycA and Glyc/SPC showed positive associations. VIP analysis highlighted tyrosine, 3-hydroxybutyrate, triglycerides, VLDL particle number and succinic acid. PCA and PLS-DA showed no clear separation between groups, suggesting that these differences were relatively subtle. Among the significant markers, detected glycoproteins (GlycA) have been recently introduced as multiorgan systemic inflammatory markers [11,12,13]. These emerging markers, detectable only by NMR spectroscopy, in combination with Apo-A1, Apo-B100, creatinine and glucose have been recognized as a powerful multiorgan inflammatory test panel. However, they have not yet been evaluated in correlation with kidney diseases. Our proteinuria signature (VLDL, TG, GlycA) reflects findings by Burghelea et al. [14], who found lipid metabolomic dysregulation in patients with tacrolimus-induced diabetes—a point worth further investigations, underlining a possible lipid dysregulation in chronic immunosuppression toxicity. The literature evaluating proteinuria evolution is limited. In different CKD cohorts, advanced disease decreased branched-chain amino acids (BCAAs) and increased gut-derived toxins. A recent study by Dahabiyeh et al. [15] found ESRD vs. CKD had much lower valine, leucine, isoleucine and higher phenolic acids (vanillic acid, dihydroxyphenyl lactic). Our proteinuria findings involved both lipid and amino acid metabolism. This differs from CKD findings but may reflect transplant status and possibly milder dysfunction.

Compared to serum, urinary metabolomic profiles exhibited weaker and less consistent associations with renal outcomes, with only limited signals related to ΔGFR and proteinuria trajectories. A multivariate analysis performed by Blázquez-Navarro et al. [16] identified 19 NMR-derived urinary spectral features predictive of 1-year eGFR confirming that urine metabolomics can reflect global GFR trends. In a similar manner, Veríssimo et al. [17] reported that amino acids at reperfusion (phenylalanine, leucine, glycine, proline, arginine) correlated with 1-year eGFR. The metabolites identified in our cohort were different, with dimethylamine and urinary creatinine showing the clearest adjusted associations with ΔGFR.

These findings suggest that metabolomic changes linked to renal outcomes are multifactorial, with ΔGFR evolution being connected to inflammatory and energy-related pathways and proteinuria progression to lipid and amino acid metabolism. These differences support the idea that distinct aspects of kidney disease may reflect partly specific biological processes.

Our study provides novel insight by integrating both baseline serum and urinary metabolomic profiles with longitudinal renal outcomes in kidney transplant patients. Rather than describing metabolomic alterations linked to a single static graft function value, this approach allowed us to explore possible early metabolomic patterns that would associate with changes in eGFR and proteinuria over a 12-month follow-up period. By linking metabolomic profiles to the trajectory of renal function, we revealed a dynamic graft evolution rather than a single static measure of graft function, and at the same time, underscored that ΔGFR and proteinuria evolution are associated with distinct but partially overlapping metabolic signatures.

### 4.1. Strengths

This study’s strengths include a well-defined cohort with paired baseline serum and urine profiles and transparent, standardized preprocessing. We applied a multitude of tests and complementary analytical approaches—correlation analysis, group comparisons, multivariable regression, and PLS-DA with VIP scoring. Reporting exact *p*-values and highlighting borderline results enhances reproducibility and transparency. It is noteworthy to mention that the low degree of missing data—1.9% in the urinary dataset—and the internal consistency of key metabolites across methods support the reliability of the observed patterns.

### 4.2. Limitations

Analytical power was partially reduced because several statistical tests were performed on smaller outcome-specific subsets (regression Δproteinuria test was performed on a set of 50 patients) having a lower ability to detect subtle effects. Running multiple testing across hundreds of metabolites increases the risk of false positives, so findings were interpreted prudently.

A lack of pooled quality-control samples limits formal assessment of analytical drift or batch effects. Supervised multivariate methods like PLS-DA can overfit without validation, and urine metabolite levels are sensitive to dilution; alternative normalization strategies could yield different results.

Another limitation derives from the lack of maintaining a screening log of patients who were excluded before enrolment or of those who declined participation, thus limiting the ability to fully report reasons for non-participation.

Given the single-center design of this study and the inclusion of a relatively stable kidney transplant cohort, the generalizability of these findings to other transplant populations such as patients with more advanced graft dysfunction, or different clinical settings, may be limited.

Next steps should include external validation in independent, large cohorts with targeted quantitative assays for key metabolites, rigorous multiple-testing correction and reporting of model validation (PLS-DA cross-validation) to assess predictive robustness. Such confirmatory studies will determine whether these metabolites can reliably anticipate renal function or proteinuria outcomes.

Residual confounding by other clinical, metabolic and treatment-related factors, including glycemic status, lipid profile and inflammatory markers, cannot be excluded.

Overall, integrated serum and urinary metabolomics revealed distinct but partially overlapping 12-month eGFR and proteinuria signatures in kidney transplant recipients.

## 5. Conclusions

### 5.1. Serum ΔGFR

Serum metabolomics showed the clearest associations with changes in eGFR. GlycA, GlycB and Glyc/SPC were associated with ΔeGFR in the adjusted models, while valine differed between patients with positive and negative trajectories. These results suggest an association between glycoprotein related inflammatory markers and changes in renal function.

### 5.2. Serum ΔProteinuria

Several serum metabolites were associated with changes in proteinuria. Isoleucine and alanine showed negative associations with Δproteinuria, while GlycA and Glyc/SPC showed positive associations in the adjusted models. These results suggest that both amino acid metabolism and inflammatory markers may be related to proteinuria evolution.

### 5.3. Urine ΔGFR

Urinary metabolites showed fewer associations with changes in eGFR. Dimethylamine and urinary creatinine were associated with ΔeGFR in the adjusted models, while citric acid showed only a borderline association. Overall, the urinary metabolic profile showed weaker associations with renal function changes than the serum profile.

### 5.4. Urine ΔProteinuria

Only a few associations were found between urinary metabolites and changes in proteinuria. Acetic acid differed between patients with improving and worsening proteinuria, but no urinary metabolite was associated with Δproteinuria in the adjusted models. Overall, the urinary metabolic profile showed limited associations with proteinuria evolution.

## Figures and Tables

**Table 1:** Demographic characteristics of included patients.

Total number of patients	174
Sex, *n* (%)	
Female	64 (36.8)
Male	110 (63.2)
Age, years: median (IQR)	49 (39–57)
Comorbidities, *n* (%):	
Diabetes mellitus	19 (10.9)
Hypertension	142 (81.6)
Heart failure	13 (7.5)
CAD	10 (5.7)
Etiology of CKD, *n* (%)	
Chronic glomerulonephritis	47 (27)
Unknown CKD etiology	40 (23)
ADPKD	23 (13.2)
Reflux nephropathy	8 (4.6)
ANCA-associated vasculitis	7 (4)
IC-MPGN	7 (4)
FSGS	5 (2.9)
CAKUT	5 (2.9)
C3G (DDD)	5 (2.9)
Other causes	27 (15.5)
Pre-transplant dialysis, *n* (%)	127 (73)
Hemodialysis only	113 (89)
Peritoneal dialysis only	11 (8.7)
Both modalities (HD and CAPD)	3 (2.3)
Dialysis vintage, months	24 (12–46)
Donor type, *n* (%)	
Deceased donor (cadaveric)	90 (51.7)
Living related donor	75 (43.1)
Living unrelated donor	9 (5.2)
Time since transplantation, years, median (IQR)	6.3 (2.8–10.4)
Maintenance immunosuppression, *n* (%)	
Tacrolimus	118 (67.8)
Cyclosporine	44 (25.3)
Mycophenolate	163 (93.7)
Corticosteroids	147 (84.5)
mTOR inhibitors	3 (1.7)
Renal biopsy performed, *n* (%)	49 (28.2)

**Table 2:** Paired comparison of biological parameters at baseline and 12-month follow-up.

Parameter	Baseline, Median (IQR)	12 Months, Median (IQR)	Change (Δ), Median (IQR)	Paired *n*	*p*-Value
Serum creatinine	1.33 (1.09–1.82)	1.33 (1.06–1.80)	−0.02 (−0.13–0.12)	170	0.982
eGFR, mL/min/1.73 m^2^	61.0 (42.5–77.8)	59.4 (45.5–79.8)	0.9 (−5.1–6.1)	170	0.552
Urea, mg/dL	49.5 (38.0–71.8)	52.0 (37.2–71.0)	2.0 (−7.0–10.0)	154	0.234
Proteinuria, g/24 h	0.00 (0.00–0.00)	0.00 (0.00–0.11)	0.00 (0.00–0.00)	162	0.351
Hemoglobin, g/dL	13.5 (12.1–14.8)	13.3 (12.1–14.7)	−0.3 (−0.8–0.5)	159	0.047
Platelets, ×10^9^/L	247 (218–291)	247 (202–288)	−2 (−33–18)	159	0.145
White blood cells, ×10^9^/L	7.69 (6.19–9.18)	8.13 (6.62–9.57)	0.22 (−0.65–1.49)	159	0.014
CRP, mg/L	2.0 (1.1–4.2)	2.5 (1.1–4.9)	0.1 (−0.5–2.1)	31	0.171
Fasting plasma glucose, mg/dL	97.0 (89.5–108.5)	93.0 (84.0–104.5)	−4.0 (−10.0–5.0)	155	0.003
Total cholesterol, mg/dL	196.0 (167.0–227.5)	197.0 (174.0–224.0)	4.0 (−17.5–22.5)	135	0.488
Triglycerides, mg/dL	118.0 (84.8–150.5)	123.5 (88.0–176.0)	4.5 (−20.5–41.5)	132	0.089
Uric acid, mg/dL	6.6 (5.6–7.4)	6.3 (5.5–7.2)	−0.1 (−1.1–0.5)	92	0.049
Sodium, mmol/L	139.0 (138.0–140.0)	139.0 (137.2–140.0)	0.0 (−1.0–1.0)	138	0.709
Potassium, mmol/L	4.30 (4.10–4.67)	4.40 (4.10–4.70)	0.10 (−0.20–0.30)	137	0.183

**Table 3:** Associations between baseline serum and urinary metabolites and 12-month renal outcomes.

Compartment	Outcome	Metabolite	Spearman Correlation	Mann–Whitney U	Adjusted Linear Regression β (95% CI); *p*	VIP > 1
Serum	ΔeGFR	GlycA	ρ = 0.133; *p* = 0.088	*p* = 0.194	15.07 (2.13 to 28.00); *p* = 0.023	No
Serum	ΔeGFR	Valine	ρ = 0.131; *p* = 0.093	*p* = 0.033	18.53 (−17.33 to 54.39); *p* = 0.309	No
Serum	ΔeGFR	GlycB	ρ = 0.114; *p* = 0.145	*p* = 0.293	32.85 (0.62 to 65.08); *p* = 0.046	No
Serum	ΔeGFR	Glyc/SPC	ρ = 0.108; *p* = 0.165	*p* = 0.552	8.05 (0.03 to 16.07); *p* = 0.049	No
Serum	ΔeGFR	Tyrosine	ρ = 0.124; *p* = 0.112	*p* = 0.052	78.84 (−28.59 to 186.27); *p* = 0.149	Yes
Serum	ΔeGFR	Lactate	ρ = 0.074; *p* = 0.345	*p* = 0.058	−0.43 (−2.89 to 2.04); *p* = 0.734	Yes
Serum	ΔeGFR	Acetic Acid	ρ = −0.146; *p* = 0.060	*p* = 0.220	−87.18 (−177.43 to 3.08); *p* = 0.058	Yes
Serum	ΔeGFR	IDL particle number	ρ = 0.085; *p* = 0.277	*p* = 0.137	−0.002 (−0.045 to 0.041); *p* = 0.924	Yes
Serum	ΔeGFR	Pyruvate	ρ = 0.079; *p* = 0.313	*p* = 0.142	26.27 (−37.69 to 90.23); *p* = 0.418	Yes
Serum	ΔeGFR	Creatine	ρ = −0.036; *p* = 0.642	*p* = 0.733	10.56 (−73.18 to 94.30); *p* = 0.804	Yes
Serum	Δproteinuria	GlycA	ρ = 0.319; *p* = 0.023	*p* = 0.179	2.95 (0.25 to 5.64); *p* = 0.033	No
Serum	Δproteinuria	Isoleucine	ρ = −0.207; *p* = 0.144	*p* = 0.564	−18.28 (−32.39 to −4.17); *p* = 0.012	No
Serum	Δproteinuria	Glyc/SPC	ρ = 0.224; *p* = 0.114	*p* = 0.120	1.74 (0.23 to 3.25); *p* = 0.025	No
Serum	Δproteinuria	Alanine	ρ = −0.175; *p* = 0.221	*p* = 0.534	−3.77 (−7.25 to −0.28); *p* = 0.035	No
Serum	Δproteinuria	Glucose	ρ = 0.130; *p* = 0.365	*p* = 0.038	0.038 (−0.114 to 0.190); *p* = 0.615	No
Serum	Δproteinuria	VLDL particle number	ρ = −0.283; *p* = 0.044	*p* = 0.131	−0.0014 (−0.0042 to 0.0014); *p* = 0.326	Yes
Serum	Δproteinuria	Triglycerides	ρ = −0.277; *p* = 0.050	*p* = 0.157	−0.0027 (−0.0060 to 0.0006); *p* = 0.106	Yes
Serum	Δproteinuria	3-Hyroxybutyrate	ρ = 0.135; *p* = 0.346	*p* = 0.504	2.18 (−0.99 to 5.35); *p* = 0.174	Yes
Serum	Δproteinuria	Succinic Acid	ρ = 0.115; *p* = 0.420	*p* = 0.259	39.02 (−182.90 to 260.94); *p* = 0.725	Yes
Serum	Δproteinuria	Tyrosine	ρ = 0.061; *p* = 0.671	*p* = 0.343	0.14 (−19.60 to 19.88); *p* = 0.989	Yes
Urine	ΔeGFR	Dimethylamine	ρ = 0.156; *p* = 0.047	*p* = 0.494	23.05 (8.06 to 38.03); *p* = 0.003	No
Urine	ΔeGFR	Creatinine	ρ = 0.117; *p* = 0.136	*p* = 0.446	0.61 (0.15 to 1.07); *p* = 0.010	No
Urine	ΔeGFR	Citric Acid	ρ = 0.096; *p* = 0.225	*p* = 0.080	4.23 (−0.28 to 8.74); *p* = 0.066	Yes
Urine	ΔeGFR	Formic Acid	ρ = −0.114; *p* = 0.148	*p* = 0.119	−0.03 (−20.22 to 20.15); *p* = 0.997	Yes
Urine	ΔeGFR	Acetic acid	ρ = 0.033; *p* = 0.679	*p* = 0.408	4.77 (−1.25 to 10.80); *p* = 0.120	Yes
Urine	Δproteinuria	Acetic acid	ρ = −0.232; *p* = 0.106	*p* = 0.031	−0.072 (−0.970 to 0.826); *p* = 0.872	Yes
Urine	Δproteinuria	Glucose	ρ = −0.217; *p* = 0.131	*p* = 0.126	−0.005 (−0.020 to 0.011); *p* = 0.535	Yes
Urine	Δproteinuria	Glycine	ρ = −0.152; *p* = 0.292	*p* = 0.142	−0.28 (−1.15 to 0.60); *p* = 0.530	Yes

Note: Spearman correlation was used to assess associations between baseline metabolite levels and continuous changes in renal outcomes, while the Mann–Whitney U test compared metabolite levels between outcome trajectory groups. Linear regression was performed separately for each metabolite and adjusted for age, sex, baseline eGFR and transplant vintage. β values represent unstandardized regression coefficients. Positive ΔeGFR and Δproteinuria values indicate improvement. VIP > 1 indicates a variable importance in projection score above 1. *p*-values are nominal and were not adjusted for multiple comparisons.

## Data Availability

The data underlying this article cannot be shared publicly due to privacy and ethical restrictions. De-identified data may be made available from the corresponding author upon reasonable request and subject to institutional approval.

## Supplementary Materials

The following supporting information can be downloaded at https://www.mdpi.com/article/10.3390/biomedicines14081875/s1: Table S1—standardized preprocessing methods and details. Figure S1—Principal component analysis of metabolomic profiling. Figure S2—Partial least squares discriminant analysis. Figure S3—Variable importance in projection. Figure S4—Heatmap of serum metabolite profiles.

## Abbreviations

The following abbreviations are used in this manuscript:

CAD	Coronary Artery Disease
CKD	Chronic Kidney Disease
ADPKD	Autosomal Dominant Polycystic Kidney Disease
ANCA	Antineutrophil Cytoplasmic Antibody
ICMPG	Immune Complex Membranoproliferative Glomerulonephritis
FSGS	Focal Segmental Glomerulosclerosis
CAKUT	Congenital Anomalies of the Kidney and Urinary Tract
C3G (DDD)	C3 Glomerulopathy (Dense Deposit Disease)
HD	Hemodialysis
CAPD	Continuous Ambulatory Peritoneal Dialysis
CRP	C Reactive Protein
