# Supplementary material for: Integrated Serum and Urinary Metabolomics Reveal Distinct Signatures of Kidney Graft Function—A Single-Center Cohort Study
Source: Biomedicines. 2026 Aug 21;14(8):1875. doi: 10.3390/biomedicines14081875 (PMC13510207; doi:10.3390/biomedicines14081875)
Supplement: Supplementary file 1 [file biomedicines-14-01875-s001.zip › biomedicines-4467510-supplementary.pdf]

Supplementary Methods: NMR sample preparation, metabolomic profiling and data processing

Further metabolomic profiling and quantification were performed using nuclear magnetic resonance (NMR) spectroscopy. In serum, 21 metabolites and 118 lipoprotein-related parameters were profiled and quantified. The investigated lipoprotein parameters included triglycerides, cholesterol, apolipoproteins A1, A2 and B100, as well as advanced profiling of the major HDL, IDL, LDL and VLDL fractions. Glycoprotein signals, including Glyc, GlycA and GlycB, and the supramolecular phospholipid signal were also investigated. NMR profiling provided up to six subfractions of free cholesterol, triglycerides, phospholipids, ApoA1, ApoA2 and ApoB100 for each major lipoprotein fraction. A full description of the lipoprotein parameters, including subfraction densities, has been previously reported[7].

Serum samples were thawed at room temperature for approximately one hour before analysis. For NMR preparation, each sample was mixed in a 1:1 ratio with phosphate buffer, gently homogenized using a plasma rotator, and transferred into 5-mm NMR tubes (Wilmad 507). The buffer consisted of Na_2HPO_4 in D_2O and included 5 mM sodium 3-(trimethylsilyl)-[2,2,3,3-d_4]-1-propionate (TSP) as a chemical shift reference, as well as NaN_3 to prevent microbial growth. All NMR measurements were acquired at 600 MHz on a Bruker Avance Neo spectrometer equipped with an inverse-detection probe and z-axis gradients. Five experiments were recorded at 310.0 K following the Bruker Biospin IVDr V.2.0 standard operating procedures: a standard ^1H spectrum, a J-resolved two-dimensional spectrum, a diffusion-filtered 1D sequence, a CPMG spectrum, and a ^1H gradient profiling sequence.

A targeted analysis of urinary creatinine and 12 urinary metabolites, including dimethylamine, alanine, glycine, proline betaine, valine, hippuric acid, citric acid, formic acid, succinic acid, acetoacetic acid, acetone and D-glucose, was also performed using NMR under comparable conditions. Urine samples were prepared by mixing urine with buffer in a 9:1 ratio, and the same NMR experiment types were collected at 300.0 K. For urine, the buffer was based on KH_2PO_4, and components were combined using vigorous vortexing. Quantification of urinary metabolites was expressed relative to urinary creatinine. Before use, the NMR tubes were checked as previously described[8].

Continuous variables were tested for normality. Quantitative variables were analysed according to their distribution and analytical role. As most metabolite distributions were non-normal, non-parametric tests were applied. Spearman correlation coefficients were used to assess associations between baseline metabolite levels and ΔGFR. Differences between patients with negative and positive ΔGFR and between patients with worsening and improving proteinuria were analysed using the Mann–Whitney U test. Linear regression models were performed to evaluate the association between baseline metabolite levels and ΔeGFR or Δproteinuria, with adjustment for age, sex, baseline eGFR and transplant vintage. For ΔeGFR, an additional analysis including calcineurin inhibitor class was performed. A p-value <0.05 was considered nominally significant. Analyses were performed using Jamovi version 2.6. Because the study was single-center and exploratory, with multiple comparisons and incomplete metabolomic datasets for some analyses, findings were interpreted cautiously. No formal correction for multiple testing was applied; therefore, results should be interpreted as exploratory.

Metabolomic data were processed and analysed using the MetaboAnalyst platform following a standardized workflow. Missing values were replaced with one-fifth of the minimum positive value, using limit-of-detection-based imputation, rather than deletion. This approach was used to approximate values below the detection threshold and to avoid bias introduced by mean substitution methods.

An interquartile range-based method was applied to reduce clutter and to eliminate non-informative variables thus removing 25% of variables. This step was performed to retain metabolites with meaningful biological variability across patients.

For multivariate analysis, data were additionally normalized by sum, followed by log10 transformation to reduce skewness and variance and autoscaling to ensure an equal contribution of

metabolites to the analysis. This process was performed in order to ensure that all metabolites contributed in an equal manner to the analysis, regardless of their absolute concentration.

Preprocessing was followed by multivariate analysis, including principal component analysis (PCA) for global variance structure exploring, partial least squares discriminant analysis (PLS-DA) to further assess group separation. We used variable importance in projection (VIP) to identify metabolite contribution to group differentiation and heatmap analysis to visualize metabolite clustering patterns. We note that PLS-DA model performance was not externally validated, therefore caution is needed when interpreting the results because a risk of overfitting may arise.

Table S1 - standardized preprocessing methods and details

Step	Choice used	Purpose / notes
Missing value handling	Left-censored LoD imputation (1/5 min positive value)	Approximates below-detection missingness.
Low-variance filtering	IQR filter; 25% filtered out	Reduces noise by removing near-constant features.
Sample normalization	Normalization by sum	Reduces sample-to-sample magnitude differences before multivariate analysis
Transformation	Log10	Reduces skew and heteroscedasticity; stabilizes variance.
Scaling	Autoscaling	Centers/scales each metabolite to unit variance to avoid dominance by high-abundance metabolites.

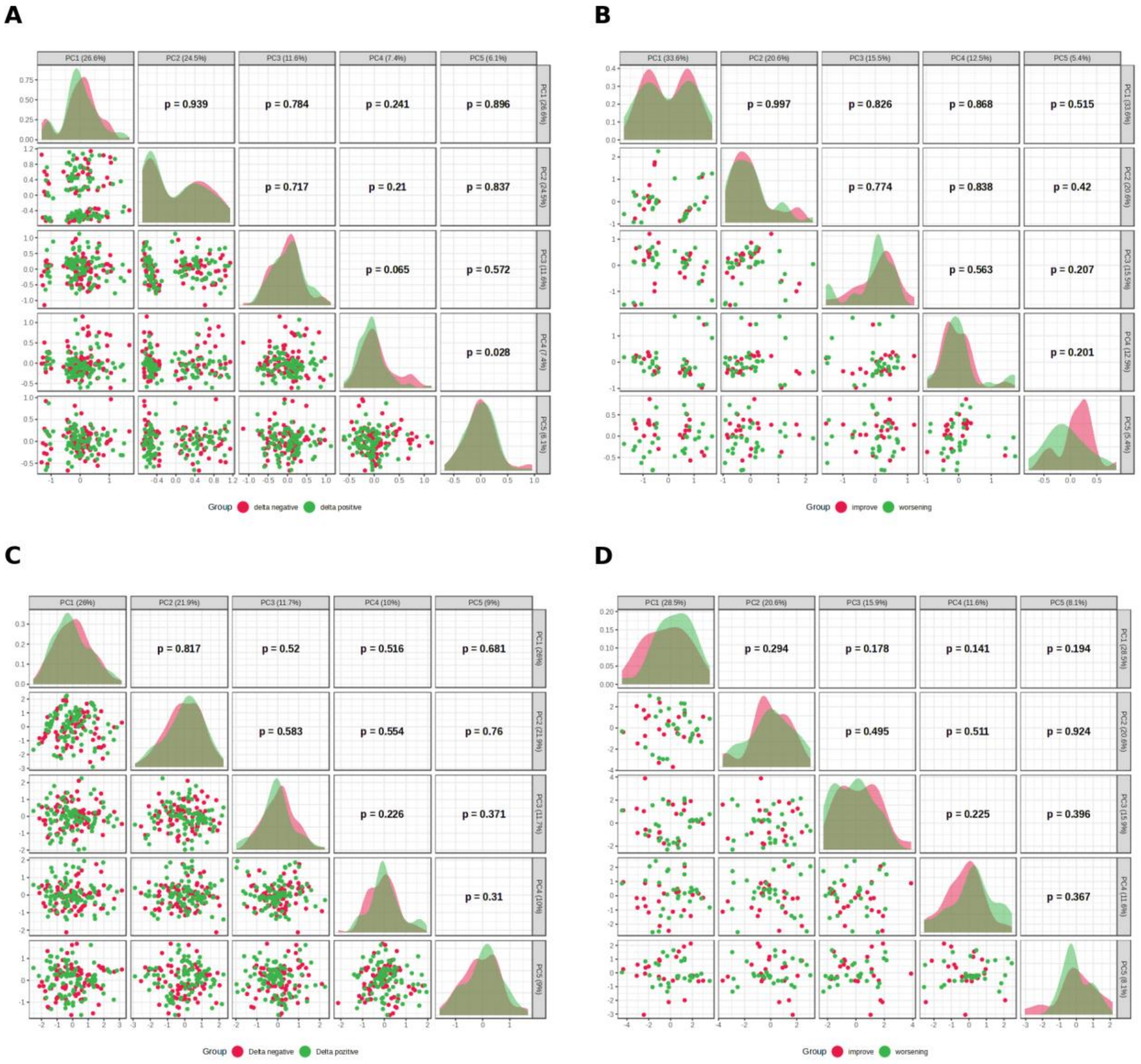

Figure S1. Principal component analysis (PCA) of metabolomic profiles according to renal outcomes. (A) Serum ΔeGFR; (B) serum Δproteinuria; (C) urine ΔeGFR; (D) urine Δproteinuria. Each point represents an individual patient.

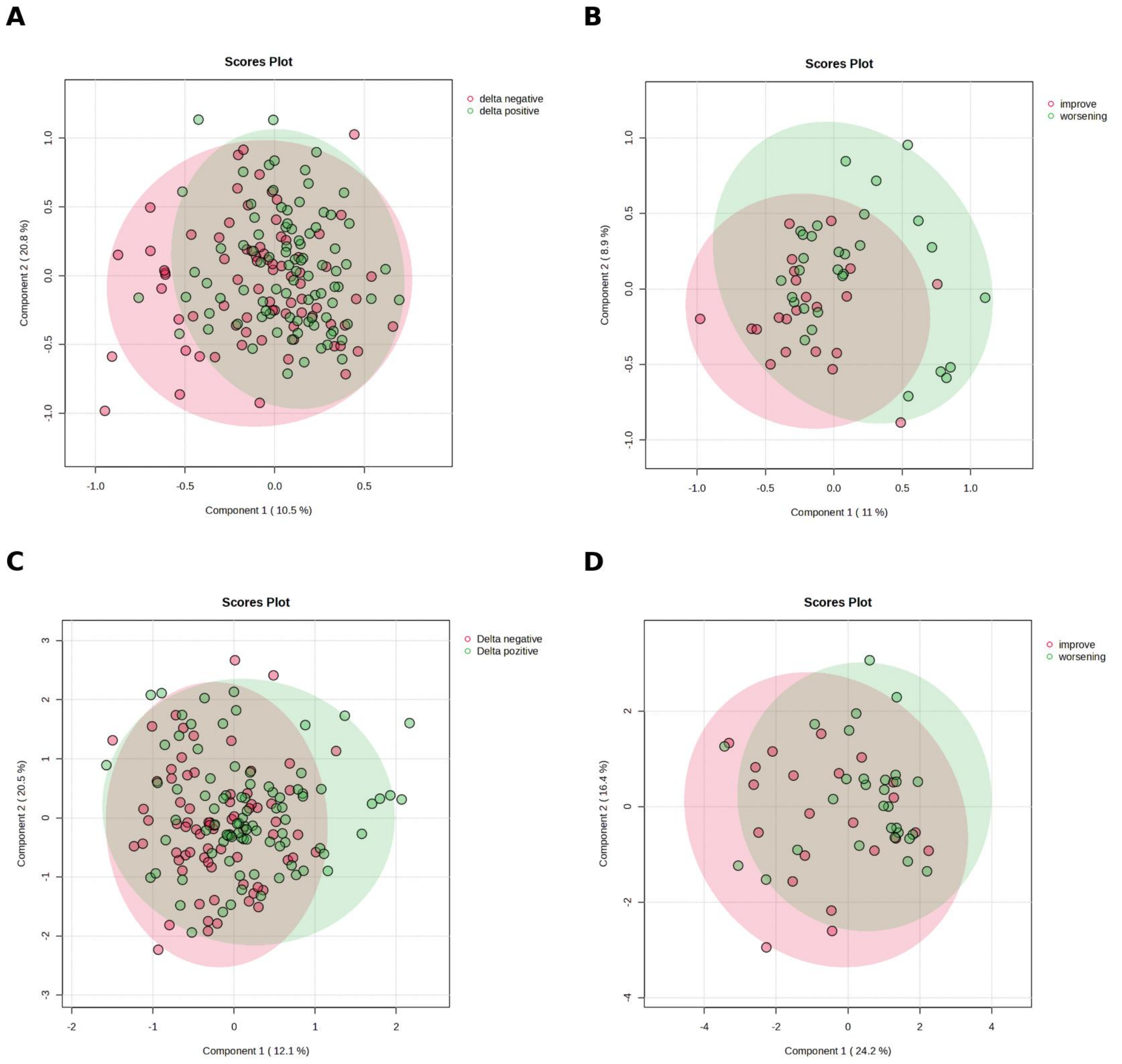

Figure S2. Partial least squares discriminant analysis (PLS-DA) score plots according to renal outcomes. (A) Serum ΔeGFR; (B) serum Δproteinuria; (C) urine ΔeGFR; (D) urine Δproteinuria. Each point represents an individual patient.

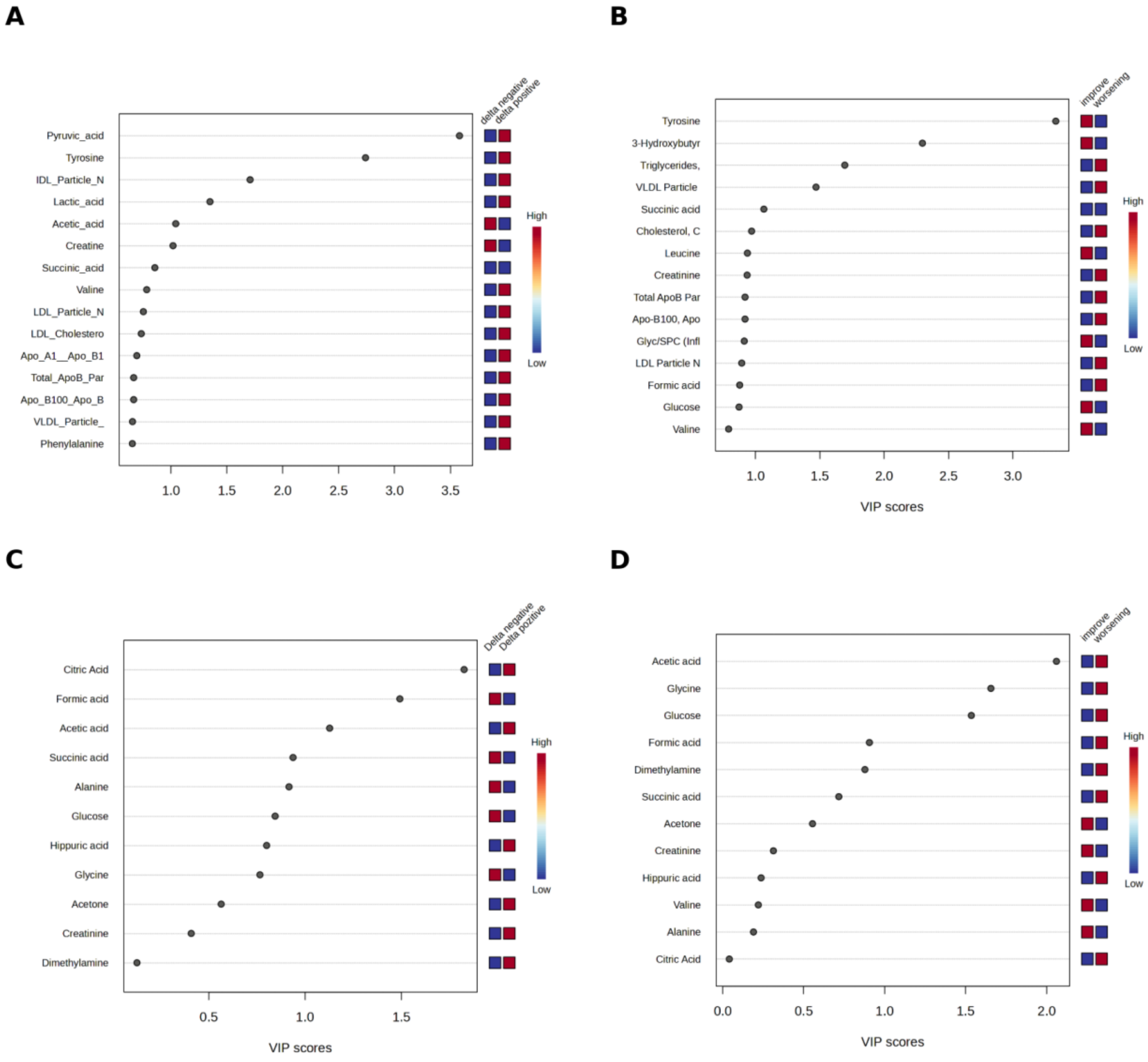

Figure S3. Variable importance in projection (VIP) plots according to renal outcomes. (A) Serum ΔeGFR; (B) serum Δproteinuria; (C) urine ΔeGFR; (D) urine Δproteinuria. Metabolites with VIP scores >1 were considered relevant contributors to group discrimination.

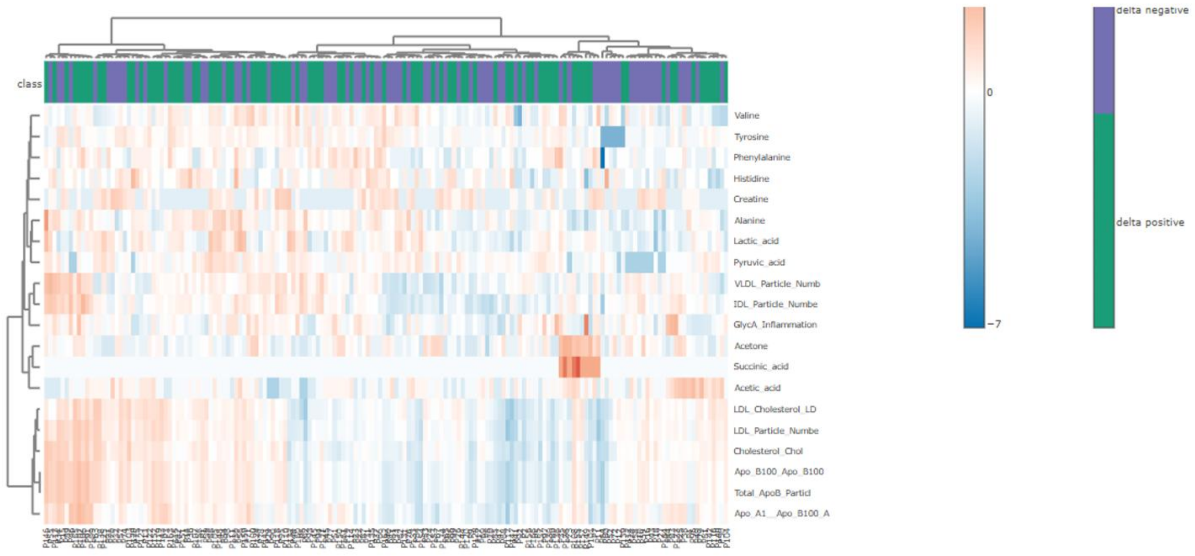

Figure S4. Heatmap of serum metabolite profiles according to ΔeGFR. Colors represent relative metabolite abundance, with hierarchical clustering applied to both samples and metabolites. No distinct clustering between groups was observed.
